# Rapamycin Ameliorates Proteinuria and Restores Nephrin and Podocin Expression in Experimental Membranous Nephropathy

**DOI:** 10.1155/2013/941893

**Published:** 2013-08-31

**Authors:** Stavros Stratakis, Kostas Stylianou, Ioannis Petrakis, Vasiliki Mavroeidi, Rafaela Poulidaki, Christina Petra, Demitrios Moisiadis, Spyros Stratigis, Eleftheria Vardaki, Lydia Nakopoulou, Eugene Daphnis

**Affiliations:** ^1^Nephrology Department, Heraklion University Hospital, 71110 Heraklion, Greece; ^2^Pathology Department, Kapodistrian University of Athens, 11527 Athens, Greece

## Abstract

*Objective*. Recent studies have shown a beneficial effect of rapamycin in passive and active Heymann Nephritis (HN). However, the mechanisms underlying this beneficial effect have not been elucidated. *Methods*. Passive Heymann Nephritis (PHN) was induced by a single intravenous infusion of anti-Fx1 in 12 Sprague-Dawley male rats. One week later, six of these rats were commenced on daily treatment with subcutaneous rapamycin 0.5 mgr/kg (PHN-Rapa). The remaining six rats were used as the proteinuric control group (PHN) while six more rats without PHN were given the rapamycin solvent and served as the healthy control group (HC). All rats were sacrificed at the end of the 7th week. *Results*. Rapamycin significantly reduced proteinuria during the autologous phase of PHN. Histological lesions were markedly improved by rapamycin. Immunofluorescence revealed attenuated deposits of autologous alloantibodies in treated rats. Untreated rats showed decreased glomerular content of both nephrin and podocin whereas rapamycin restored their expression. *Conclusions*. Rapamycin monotherapy significantly improves proteinuria and histological lesions in experimental membranous nephropathy. This beneficial effect may be mediated by inhibition of the alloimmune response during the autologous phase of PHN and by restoration of the normal expression of the podocyte proteins nephrin and podocin.

## 1. Introduction

Membranous nephropathy (MN) is a common cause of nephrotic syndrome (NS), accounting for approximately 20% of cases in Caucasians [1]. MN is characterized by thickening of the glomerular basement membrane (GBM) and deposition of immune complexes and complement on its subepithelial aspect. 

Spontaneous complete or partial remission of proteinuria occurs in 5–32% and 25–40%, respectively, at five years [2–5]. The probability of end-stage renal disease (ESRD) in untreated patients is approximately 15% at five years, 35% at 10 years, and 40% at 15 years [2–4, 6]. Due to the relatively benign clinical course, immunosuppressive agents are considered only in patients at risk of progressive disease or with severe symptomatic NS [2, 7, 8]. 

Recent evidence suggests that the majority of patients with idiopathic MN have circulating antibodies against phospholipase A2 receptor (PLA2R), which is present on podocytes, as is megalin in rat models of MN [9]. Similarly, neutral endopeptidase has been found as the target antigen in newborns' podocytes with alloimmune neonatal membranous nephropathy [10] and cationic bovine serum albumin as a planted antigen in early childhood MN [11]. Additional circulating autoantibodies against human podocytic antigens have recently been described [12]. It is speculated that as a result of podocyte injury by complement, various intracellular proteins and cryptic epitopes may be exposed, thus inducing “a second wave of immunisation” [13, 14].

Heymann Nephritis is a faithful experimental model of the disease that has been extensively studied since first described by Heymann et al. in 1959 [15]. The active model of HN is induced by immunization of Lewis rats with preparations of brush-border proteins. The passive model of HN (PHN) is induced by a single i.v. injection of heterologous anti-brush border antiserum (anti-Fx1A) that produces heterologous IgG subepithelial deposits within hours to days. Proteinuria occurs in almost all animals within five days. This “heterologous phase” is followed, two weeks later, by an “autologous phase” during which rat IgG antibodies are produced against the heterologous IgG. The autologous IgG alloantibodies are also deposited at the subepithelial space, inducing a further increase in proteinuria [16]. The second (autologous) phase of PHN mimics idiopathic MN because during the autologous phase there is production of autoantibodies (similar to human disease), against a planted exogenous antigen (similar to cationic bovine serum albumin in humans) but also against neoantigens that are exposed in the subepithelial space during the initial injury (again similar to the second wave of immunization that is believed to happen in human idiopathic MN).

Therefore it is the autologous phase of passive HN that shares the same pathophysiological mechanisms to those recently identified in idiopathic MN in humans. 

To date the therapeutic approach has not changed substantially. The monthly alteration of cyclophosphamide or cyclosporine and corticosteroids remains the standard therapy for severe and persisting proteinuria. Given the significance of IgG antibodies in MN, strategies to target B lymphocytes and antibody formation may be effective in inducing remission of the NS [14]. Indeed there is recent evidence that anti-CD20 antibody administration can effectively treat patients with idiopathic MN [17, 18]. 

The immunosuppressive effect of rapamycin was first attributed to the inhibition of cytokine-induced proliferation and clonal expansion of T cells. More recently, it has become evident that rapamycin (in contrast to tacrolimus and cyclosporine) inhibits the proliferation of B cells [19] and restricts B cells capable of producing immunoglobulins [20]. 

Bonegio et al. demonstrated that low dose rapamycin ameliorated proteinuria in experimental PHN and limited tubulointerstitial inflammation and interstitial fibrosis in association with reduced expression of proinflammatory and profibrotic genes [21]. The beneficial effects of rapamycin have also been observed in active HN [22]. Here we tried to investigate more specific effects of rapamycin, beyond the known antifibrotic ones. In particular we examined the effect of rapamycin on podocytes architecture and slit diaphragm proteins, as well as on the deposition of pathogenic autoantibodies that coincides with the autologous phase of PHN.

## 2. Materials and Methods

### 2.1. Experimental Design

Eighteen male Sprague-Dawley rats (Pasteur Institute, Athens, Greece) were used in this study. The experiment was carried out in accordance with current legislation on animal experiments in the European Union and approved by our institution's Safety and Ethics Committee for Animal Research. All animals were housed in a room with 12 h light/12 h dark cycle, constant temperature of 22°C, and had free access to standard diet and water. PHN was induced in 12 rats by a single i.v. infusion of 0.5 mL sheep anti-Fx1 per 100 gr of body weight. Anti-Fx1 antiserum was kindly provided by Dr. Kerjaschki. Rats were anesthetized by intraperitoneal infusion of Ketamine 67 mg/kg and Xylazine 10 mg/kg. 

One week after anti-Fx1 infusion all rats became proteinuric. Six of them were randomly selected to commence daily subcutaneous injections of rapamycin (Sigma, St Louis, MO, USA) at a dose of 0.5 mgr/kg (PHN-Rapa group). Another six rats with PHN were given subcutaneously only the rapamycin solvent (DMSO) and served as the passive HN proteinuric group (PHN group). The remaining six, age and weight matched healthy rats without PHN, received only DMSO and served as the healthy control group (HC group). Urine collections were performed weekly in metabolic cages (Tecniplast, Italy). Body weight was also determined weekly and rapamycin dose was adjusted accordingly. All animals were sacrificed 7 weeks after anti-Fx1 administration.

### 2.2. Isolation of Glomeruli

Glomeruli were isolated by differential sieving by utilizing sieves (Retsch, Haan, Germany) of different pore sizes: 150 *μ*m, 106 *μ*m, and 75 *μ*m. Isolated glomeruli were retained on the bottom screen of 75 *μ*m pore size. Purity of the glomerular isolate was estimated to be >95%. After several washings with PBS, glomeruli were collected and centrifuged for 4 min at 1200 r.p.m. The pellet was homogenized in RIPA-buffer containing protease inhibitors and was stored in −80°C till analysis. 

### 2.3. Western Blot (WB) Analysis

Kidney cortex tissue was homogenized in RIPA-buffer containing protease inhibitors (Roche Diagnostics, Hellas, SA). Forty *μ*g of glomerular lysate was electrophorized per lane on 7.5% SDS-gels. The proteins were transferred electrophoretically on nitrocellulose membranes (Schleicher & Schuell BioScience GmbH, Germany). Membranes were blocked with 5% BSA (Sigma-Aldrich) in TBS-1X Tween-20 0.1% and incubated overnight at 4°C with guinea pig nephrin pAb (1 : 500) (Progen Biotechnik GmbH, Germany), rabbit podocin pAb (1 : 500) (Abcam, Cambridge, UK), and with mouse anti-actin mAb (1 : 3000) (C4; Chemicon International, Temecula, CA). Appropriate HRP-linked antibodies (Cell Signaling Technology) were applied for 60 minutes at room temperature. Signal was detected using appropriate chemiluminescence reagent (Amersham Biosciences, GE Healthcare, UK). Bands were normalized to actin expression. Image-J (NIH, MD, USA) densitometry analysis system was used for measurements.

### 2.4. Real Time RT-PCR (qRT-PCR)

Renal tissue was homogenized in Trizol Reagent (Life Technologies; Gibco BRL, Paisley, UK). One *μ*g of total RNA was reverse transcribed (Superscript-II; Gibco) and amplified by RT-PCR. Products were normalized according to glyceraldehyde-3-phosphate dehydrogenase (GAPDH) expression. Measurements were performed using the ABI-Prism 7000 System (Applied Biosystems; California, USA). iTaq SYBR-Green Supermix with ROX (Bio-Rad) was used for the reactions. Results were normalized to GAPDH and analysis was performed using the 2^−ΔΔCt^ method. All samples were tested in duplicate.

### 2.5. Microscopy Studies

Left kidney sections were fixed in neutral formalin and examined by a renal pathologist (L. Nakopoulou) who was blinded to the group assignment. 

For immunofluorescence (IF) studies left kidney sections were embedded in OCT compound (Sakura Finetek USA, Inc), snap frozen in liquid nitrogen, and stored in −80°C until examination. Five-micrometer thick cryosections were incubated overnight with the same primary antibodies used in western blot. Dilution for nephrin was 1 : 250 and for podocin was 1 : 100. Secondary antibodies used included Alexa Fluor-488 conjugated goat anti-rabbit or anti-guinea pig or anti-rat IgG at 1/1000 (Molecular Probes, Inc). RNAse (Sigma) diluted in BSA1% PBS Tween1X (1 : 500) was applied for 30 min and then samples were incubated with propidium iodide 1 : 1000 (Sigma) for 5 minutes. At least 30 glomeruli were examined per animal. The intensity of the fluorescence was scored on a scale of 0 to 3+, where 0 = absent, 1+ = mild, 2+ = moderate, and 3+ = strong staining.

For EM studies left kidney sections were processed as usual and examined under a transmission electron microscope (JEM100CX-II; JEOL Inc., Tokyo, Japan). Twenty random glomeruli were examined for each mouse. Microphotographs were analyzed using the Digital Micrograph software (Gatan GmbH, Munchen, Germany). The entire curved length of the GBM of all open capillary loops (loop length, LL) and the number of foot processes (FPN) overlying capillary loops were measured. The foot process width (FPW) in each loop was calculated using the formula: FPW = (*π*/4 × LL)/FPN [23]. The foot process density (FPD) in each loop was measured using the formula: FPD = FPN/LL.

### 2.6. Measurement of Proteinuria, Serum Creatinine, and Rapamycin Levels

Urinary protein concentration was determined with the Bio-Rad protein assay (Bio-Rad, Hercules, CA). Serum creatinine levels were measured at sacrifice by an autoanalyzer (Olympus 600, Tokyo, Japan). Rapamycin blood levels were determined by EIA in whole blood (Imx Analyzer, Abbott Lab, USA).

### 2.7. Statistical Analysis

Analysis of variance (ANOVA) was performed to compare serum creatinine and IF scores between groups. Continuous variables are expressed as mean ± SE. Repeated measures analysis of variance was used to compare the weekly measurements of proteinuria and body weight throughout the study. Independent samples Kruskal-Wallis and median tests were used for nonparametric comparisons. Differences were considered significant for a *P* less than 0.05 (two tailed). SPSS19-IBM software was used for statistical analysis.

## 3. Results

### 3.1. Clinical and Biochemical Characteristics

**Figure 1:**
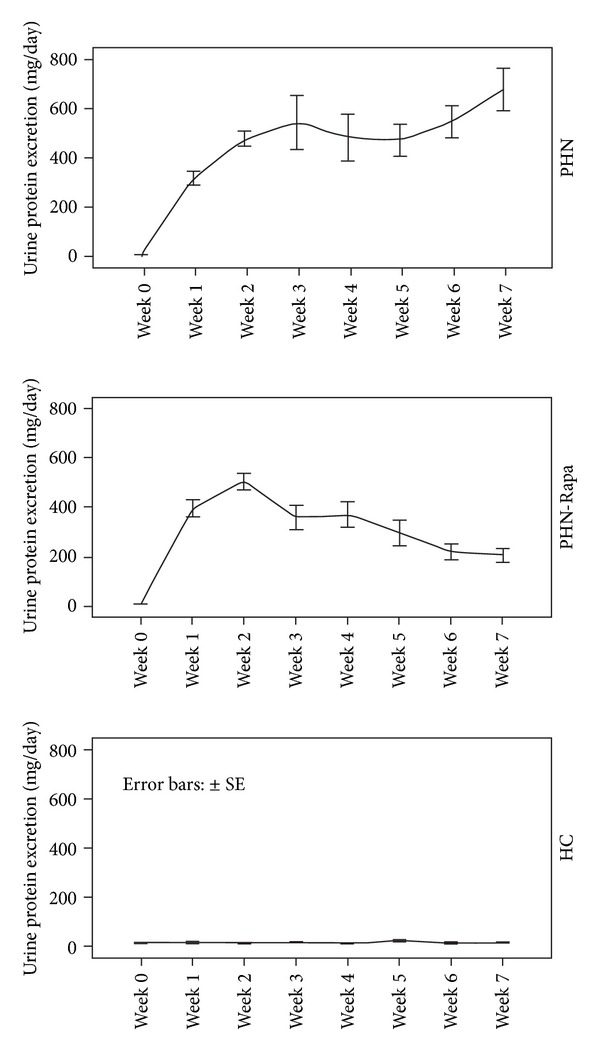
24-hour urine protein excretion in study groups. Rapamycin was administered at week one in the PHN-Rapa group resulting in gradual amelioration of proteinuria in contrast to the PHN group in which proteinuria continued deteriorating.

The 24-hour urinary protein of both PHN and PHN-Rapa groups increased at nephrotic levels at day 7 and remained so until the 2nd week after nephritis induction. Thereafter proteinuria began to decline in the PHN-Rapa group while it continued deteriorating in the PHN group. At the end of the study (week 7) urine protein levels in the PHN-Rapa group were 1/3 of those in the PHN group (*P* = 0.007, by repeated measures ANOVA). Although proteinuria in the PHN-Rapa group declined continuously, it did not reach the urine protein levels of HCs at the time of sacrifice (Figure 1).

Rats in the PHN-Rapa group did not increase BW at the same pace as the other groups. At the end of the study their BW was 66% and 45% of that in the PHN and HC groups (Table 1). The ratio of renal to body mass at the end of the study was higher (*P* < 0.01) in the PHN group (0.013) compared to PHN-Rapa group (0.010) and HC group (0.009). Serum creatinine, total protein, and total cholesterol are presented in Table 1. Serum creatinine levels were higher in the HC group due to the higher body weight in this group at the end of the study. Serum total protein and albumin levels in the PHN-Rapa group were significantly higher as compared to the PHN group but did not reach those of HCs (Table 1). The trough rapamycin levels averaged at 12.5 ± 0.76 ng/mL in treated rats.

### 3.2. Photon Microscopy, Immunofluorescence, and Electron Microscopy

**Figure 2:**
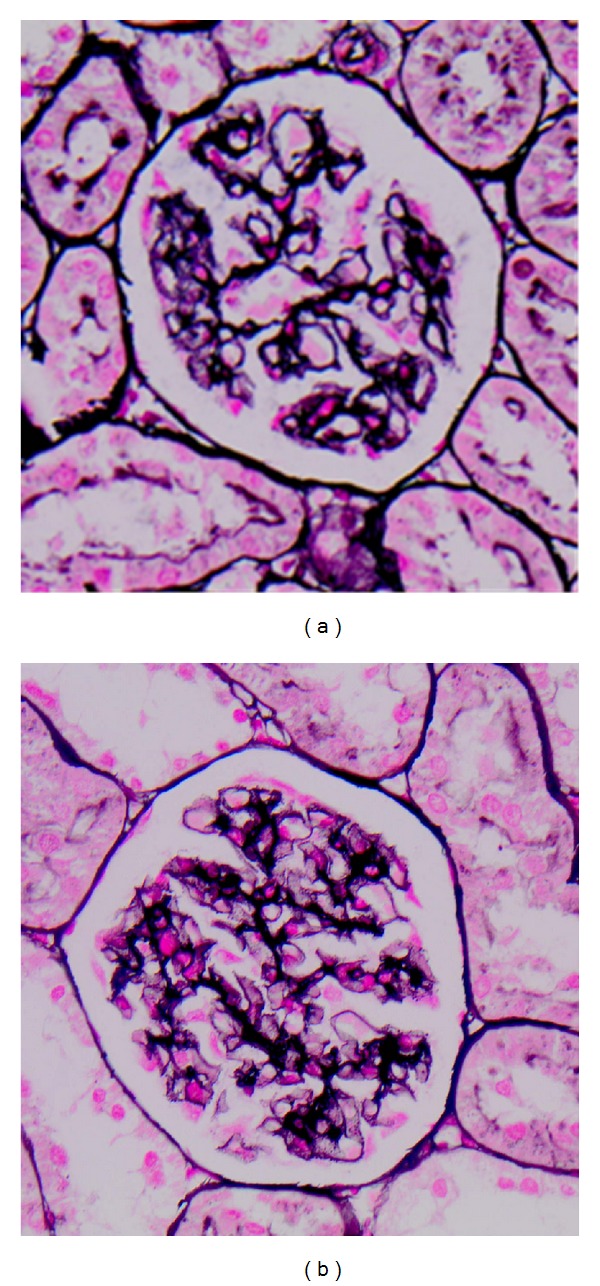
Photon microscopy. (a) Passive Heymann Nephritis. Moderate to severe irregular thickening of glomerular capillary basement membranes (Silver Methenamine, ×400). (b) Passive Heymann Nephritis after rapamycin administration. Mild to moderate thickening of glomerular capillary basement membranes (Silver Methenamine, ×400).

**Figure 3:**
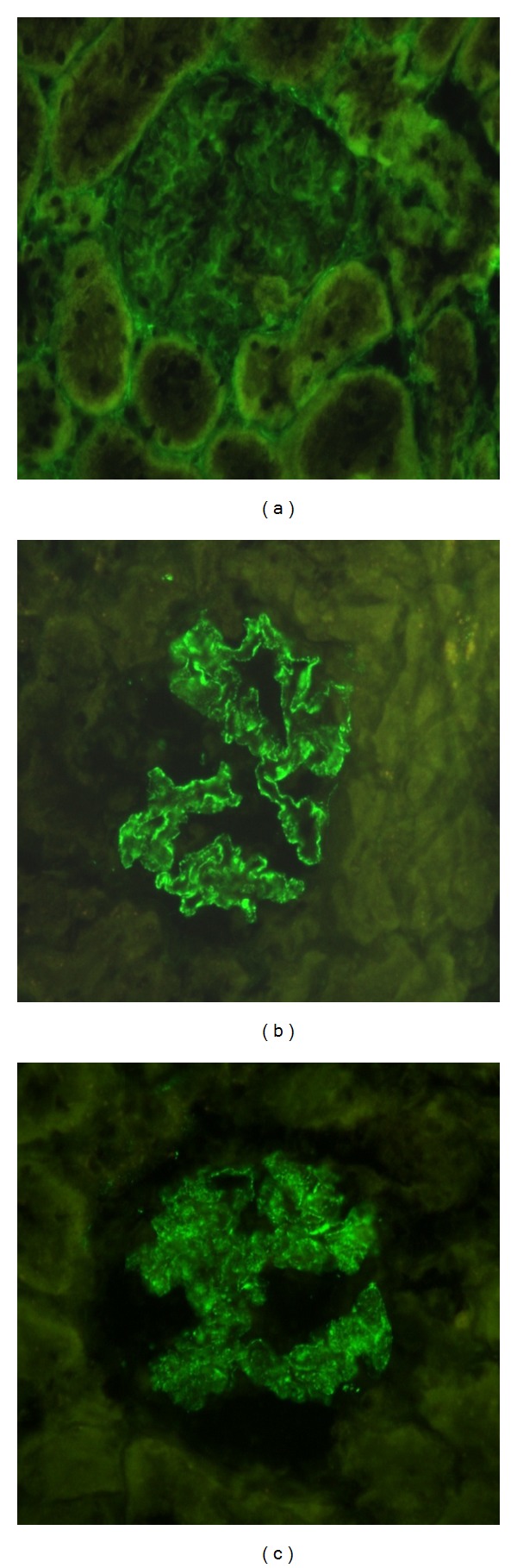
Immunofluorescence staining with anti-Rat IgG. (a) Healthy controls showed absence of staining. Original magnification ×400. (b) Passive Heymann Nephritis; glomeruli showed intense (3+) granular and irregular fluorescence along the capillary walls (×400). (c) Passive Heymann Nephritis after rapamycin administration; staining was significantly attenuated (1+ to 2+) in almost all glomeruli (×400).

**Figure 4:**
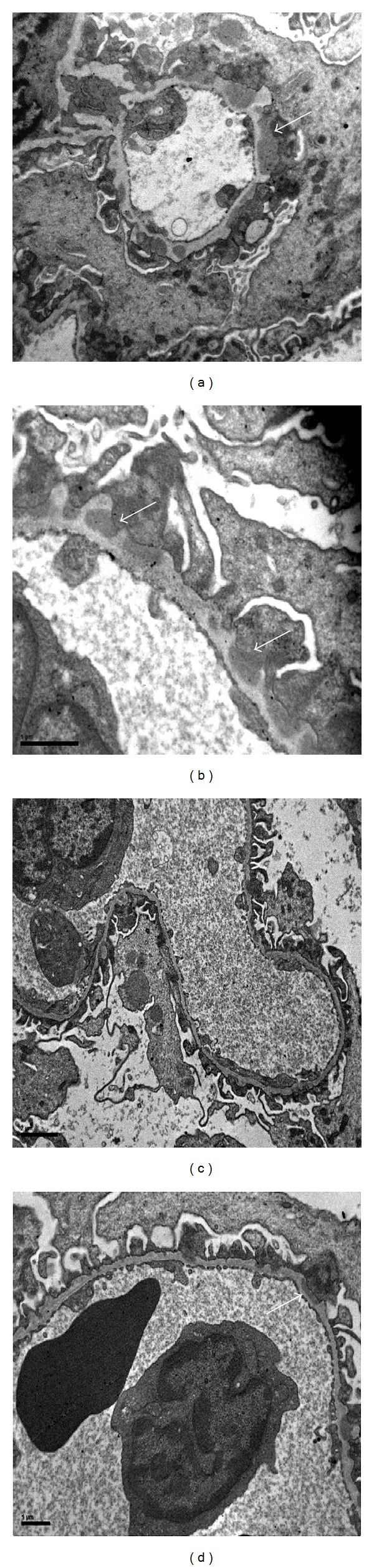
Electron microscopy. (a) Passive Heymann Nephritis. Massive subepithelial deposits (white arrow) with severe fusion of podocyte foot processes; original magnification ×12 k. (b) Passive Heymann Nephritis. Subepithelial deposits in higher magnification (white arrows); original magnification ×26 k. (c) Passive Heymann Nephritis after rapamycin administration. The deposits and the podocytic injury were attenuated compared to the PHN group; original magnification ×8 k. (d) Passive Heymann Nephritis after rapamycin administration (higher magnification ×20 k). Small subepithelial deposit (white arrow).

After staining with silver methenamine the glomeruli in the PHN group revealed moderate to severe thickening of the GBM, while rapamycin treated rats displayed less severe histological lesions with only mild or moderate thickening (Figure 2). Cryosections stained for anti-Rat IgG showed intense (3+) granular and irregular fluorescence along the glomerular capillary walls of the PHN group, whereas staining was attenuated (1+ to 2+) in the PHN-Rapa group and absent in the control group (Figure 3). Electron microscopy showed massive subepithelial deposits in almost all capillary loops in the PHN group with severely affected podocytes (Figure 4). In particular the harmonic mean and the median value of FPW were 830.78 nm and 789.26 nm, respectively, while the mean foot process density per *μ*m of GBM length (FPD) was 0.94 ± 0.42. In the PHN-Rapa group the deposits and the podocytic injury were markedly attenuated compared to the PHN group (harmonic mean of FPW 613.3 nm, median 654.1 nm, and FPD 1.28 ± 0.9; all *P* < 0.001). Despite this improvement, the respective values in the HC were much lower (harmonic mean FPW 349 nm, median FPW 355 nm, and mean FPD 2.24 ± 0.43; all *P* < 0.001 as compared to other groups).

### 3.3. Nephrin and Podocin Expression

**Figure 5:**
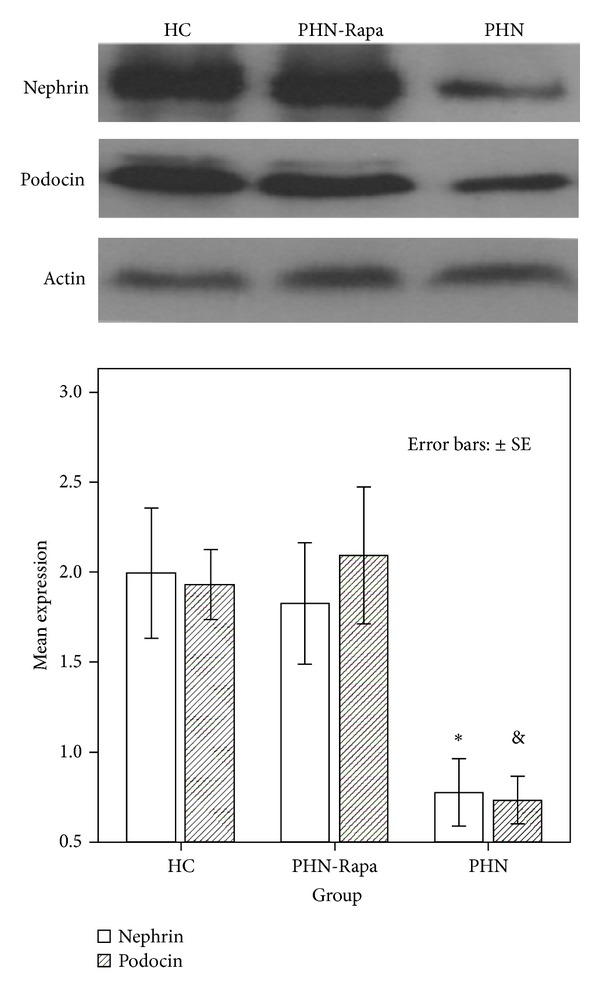
Western blot analysis for nephrin, podocin, and actin in glomerular lysates. HC: healthy controls; PHN: Passive Heymann Nephritis; PHN-Rapa: Passive Heymann Nephritis after rapamycin administration. Bars represent nephrin (white columns) and podocin (shaded columns) protein content corrected to actin concentration. *P = 0.01, ^&^ *P* = 0.007. Error bars: ±SE.

By WB the levels of nephrin and podocin protein levels in glomerular lysates were significantly lower (all *P* < 0.05) in the PHN group as compared to the HC and PHN-Rapa groups. In the PHN-Rapa group, nephrin and podocin levels were similar to HCs (Figure 5).

**Figure 6:**
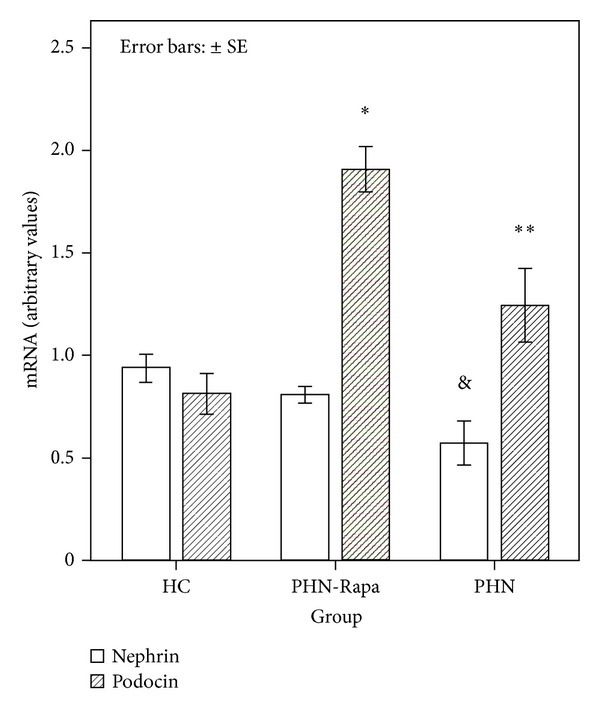
Real time PCR for nephrin and podocin mRNA in glomerular lysates. HC: healthy controls; PHN: Passive Heymann Nephritis; PHN-Rapa: Passive Heymann Nephritis after rapamycin administration. Bars represent nephrin mRNA (white columns) and podocin mRNA (shaded columns) corrected to GAPDH mRNA concentration. *P < 0.001, ^&^ *P* = 0.011, **P = 0.038. Error bars: ±SE.

By RT-PCR the expression of nephrin mRNA was lower in the PHN group compared to HC and PHN-Rapa groups (*P* = 0.011 and *P* = 0.039, resp.; Figure 6). In contrast podocin mRNA was increased in the PHN and PHN-RAPA groups as compared to HCs (Figure 6).

**Figure 7:**
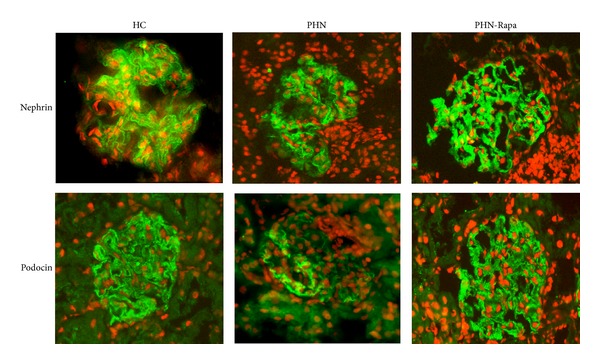
Immunofluorescence staining for nephrin and podocin. HC: healthy controls; PHN: Passive Heymann Nephritis; PHN-Rapa: Passive Heymann Nephritis after rapamycin administration. Nuclei have been stained with propidium iodide (red). Intense (3+) linear staining in the HC and PHN-Rapa groups in contrast to the PHN group which presented attenuated and irregular pattern of staining for both nephrin and podocin (middle panel).

Immunofluorescence for nephrin and podocin showed intense (3+) and regular linear staining in all examined glomeruli in the HC group. Normal staining for both proteins was also evident in the majority of glomeruli (72.8%) in the PHN-Rapa group. On the contrary, staining for nephrin and podocin was irregular and attenuated (<3+) in almost all glomeruli in the PHN group (Figure 7).

## 4. Discussion

Rapamycin treatment has shown either protective [21, 22, 24–31] or untoward [32–36] results in various forms of experimental or human kidney disease. In summary, rapamycin displays dual opposing effects, with proteinuria and podocyte damage aggravation in the toxicoimmunological glomerular models and a nephroprotective effect in the chronic inflammatory glomerulotubulointerstitial models [37]. Rapamycin inhibits the proliferation of both T and B cells [19] and reduces the number of B cells capable of producing immunoglobulins in contrast to cyclosporine and tacrolimus [20]. Rapamycin can also promote the generation of regulatory T cells which suppress the immune system and control autoimmunity [38]. These combined properties make sirolimus an attractive agent for the treatment of autoimmune diseases such as MN. 

In the present study rapamycin was given after induction of HN when severe proteinuria, and by inference histological lesions, had already been established. Nevertheless, rapamycin was able to abrogate the second rise of proteinuria during the autologous phase. This clinical result was escorted by significant alleviation of the histological lesions. More precise podocyte indices such as FPW and FPD were markedly improved by rapamycin whereas the expression of slit diaphragm proteins nephrin and podocin was almost completely restored. It is interesting that in the PHN group, podocin mRNA levels were increased, nephrin mRNA levels were decreased, and the respective protein levels were both decreased. This discrepancy implies that nephrin decreased owing to suppressed translation, whereas the decreased podocin levels may be due to loss or destruction of the protein with a compensatory increase in podocin mRNA levels. Residual histological lesions seen in the PHN-Rapa group should be attributed to the initial insult during the heterologous phase when the drug had not been given yet. The attenuation of anti-Rat IgG staining in IF indicates that rapamycin blocked the production of pathogenic autologous alloantibodies (possibly via its B-cell inhibitory effects) that are responsible for the second boost of proteinuria, resulting thus in the gradual resolution of the NS. These results are in line with previous studies in animal models of MN [21, 22] and offer further insights into possible mechanisms for the therapeutic effect of rapamycin in experimental MN. 

## 5. Conclusions

Rapamycin significantly improves proteinuria and histological lesions during the autologous phase of PHN, an effect that may be mediated by inhibition of the autoimmune response and by restoration of the normal expression of the podocyte proteins nephrin and podocin. If our results are confirmed by future studies, rapamycin may prove to be an effective treatment for MN.

## Figures and Tables

**Table 1:** Clinical and biochemical characteristics of groups under study.

	Group			*P*		
	PHN	PHN-Rapa	HC	PHN versus PHN-Rapa	PHN versus HC	PHN-Rapa versus HC
Initial body weight (gr)	170 ± 2.8	169 ± 5.2	180 ± 2.1	0.9	0.12	0.1
Final body weight (gr)	335.7 ± 7.3	268.3 ± 12.1	432.5 ± 9.2	<0.001	<0.001	<0.001
Kidney mass (gr)	4.3 ± 0.3	2.8 ± 0.2	4.04 ± 0.02	0.001	0.47	0.009
Kidney mass over body weight	0.013 ± 0.0002	0.010 ± 0.0004	0.009 ± 0.0003	0.013	0.002	0.27
Serum creatinine (mg/dL)	0.30 ± 0.001	0.31 ± 0.03	0.40 ± 0.001	0.55	0.006	0.016
Serum total protein (mg/dL)	5.91 ± 0.14	6.33 ± 0.12	6.77 ± 0.075	0.032	0.001	0.04
Serum albumin (mg/dL)	2.8 ± 0.09	3.1 ± 0.1	3.6 ± 0.04	0.03	0.002	0.04
Serum cholesterol (mg/dL)	187.5 ± 21	313.3 ± 53	66 ± 5	0.03	0.05	0.001
